# A pipeline for estimating human attention toward objects with on-board cameras on the iCub humanoid robot

**DOI:** 10.3389/frobt.2024.1346714

**Published:** 2024-10-17

**Authors:** Shiva Hanifi, Elisa Maiettini, Maria Lombardi, Lorenzo Natale

**Affiliations:** Humanoid Sensing and Perception Group, Istituito Italiano di Tecnologia, Genoa, Italy

**Keywords:** attention, gaze estimation, learning architecture, humanoid robot, computer vision, human–robot scenario

## Abstract

This research report introduces a learning system designed to detect the object that humans are gazing at, using solely visual feedback. By incorporating face detection, human attention prediction, and online object detection, the system enables the robot to perceive and interpret human gaze accurately, thereby facilitating the establishment of joint attention with human partners. Additionally, a novel dataset collected with the humanoid robot iCub is introduced, comprising more than 22,000 images from ten participants gazing at different annotated objects. This dataset serves as a benchmark for human gaze estimation in table-top human–robot interaction (HRI) contexts. In this work, we use it to assess the proposed pipeline’s performance and examine each component’s effectiveness. Furthermore, the developed system is deployed on the iCub and showcases its functionality. The results demonstrate the potential of the proposed approach as a first step to enhancing social awareness and responsiveness in social robotics. This advancement can enhance assistance and support in collaborative scenarios, promoting more efficient human–robot collaborations.

## 1 Introduction

Any face-to-face interaction between two people is characterized by a continuous exchange of social signals, such as gaze, gestures, and facial expressions. Such non-verbal communication is possible because interacting individuals can see, perceive, and understand the social information enclosed in cues. In this study, we prioritize eye gaze, a critical social cue, because it plays a pivotal role in many mechanisms of social cognition, for example, joint attention, regulating and monitoring turn-taking, signaling attention, and intention. Neuropsychological evidence highlighted the close relationship between gaze direction and attention, indicating that gaze functions are actively involved and influenced by spatial attention systems (Allison et al., 2000; Pelphrey et al., 2003). For example, it is more likely that the gaze is directed toward an object rather than toward empty space.

In this context, a robot’s ability to determine what a human is looking at (e.g., an object) has numerous practical implications across various domains. In social robotics, it enhances a robot’s social awareness and responsiveness, making interactions more natural and context-appropriate (Babel et al., 2021; Holman et al., 2021). This includes recognizing a person’s preferences based on their gaze and improving collaboration in settings like industry or home by understanding human attention (Kurylo and Wilson, 2019).

This research report represents the initial milestone in our ongoing study aiming at interpreting human intent during human–robot collaboration. We introduce a novel HRI application utilizing computer vision to enable robots to detect the object a human partner is gazing at. This application sets the baseline for forthcoming advancements in our research. Our proposed system combines an online object detection algorithm (Ceola et al., 2021; Maiettini et al., 2019a) with gaze tracking technologies, providing the robot with online information about the objects that capture the human’s attention. This integration grants the robot enhanced cognitive ability to perceive and interpret human gaze accurately in its environment. This could be the initial step in enabling the robot to achieve conscious joint attention with the human partner (Chevalier et al., 2020).

The main contributions are as follows:

• We propose a pipeline to detect the target of human attention during an interaction with a robot. This leverages face detection, human attention prediction, and online object detection to detect the object the human focuses on.

• We present the *ObjectDetection* dataset collected with the humanoid iCub (Metta et al., 2010), where 10 participants gaze at different objects placed randomly on a table in front of the robot, including annotations of ground truth gaze target and object bounding boxes.

• We perform an experimental analysis of the proposed pipeline to evaluate its effectiveness in the considered HRI setting. We use the collected dataset to do that, and we study the performance of the components of the system.

• Finally, we deploy the system on the iCub robot. A video is submitted as Supplementary Material.

## 2 Related work

The problem of endowing robots with the capability to comprehend human behavior, particularly the social cue of the gaze, has been studied in the literature. In this regard, the human line of sight, which consists of two main components—the head pose and the orientation of the eyes within their sockets (eyegaze) (Wang and Sung, 2002)—offers critical information for predicting human attention and intention. Although extended literature addresses the use of egocentric gaze data from external wearable devices [e.g., head-mounted eye trackers (Admoni and Srinivasa, 2016) and chest-mounted cameras (Furnari et al., 2017; Bertasius et al., 2016)] or using a geometric approach to estimate gaze [where the eyes and pupils need to be clearly visible in the image (Palinko et al., 2015)], our study upholds a naturalistic HRI setting by avoiding external devices utilizing a third-person view and positioning the human partner at a distance from the robot.

In this context, the gaze problem is addressed following two different strategies: 1) gaze estimation (i.e., estimating the gaze vector or mutual gaze events) and 2) gaze attention prediction (i.e., understanding where the human is visually attending in terms of a saliency map).

Following the *gaze estimation strategy*, Wang and Sung (2002) employ zoom-in iris imaging to estimate eye gaze from a single eye. They integrate head pose and eye gaze determination for enhanced accuracy. Other works focus on human gaze estimation using a 2D/3D vector. For example, the use of the CNN architecture to estimate the 2D gaze vector is proposed by Athavale et al. (2022). This system extracts features from only one eye and is especially useful in real-world conditions where the human face can be partially obscured. In this regard, Fischer et al. (2018) propose a novel dataset of varied gaze and head pose images in a natural environment, addressing the issue of ground truth annotation by measuring head pose using a motion capture system and eye gaze using mobile eye-tracking glasses. Examples of predicting a 3D gaze vector can be found in Cheng et al. (2020) and Ververas et al. (2022). Specifically, Ververas et al. (2022) propose an architecture to estimate the vector of the gaze direction from the reconstructed dense 3D eyeball meshes. Cheng et al. (2020), instead, propose a combination of a regression and an evaluation network able to exploit the asymmetry between the left and right eye. Additionally, Lombardi et al. (2022a) propose a learning architecture to detect mutual gaze events. This study underscores the significance of mutual gaze as a vital social cue in face-to-face interactions, indicating the readiness of interacting partners.

The *gaze following* problem was addressed by Recasens et al. (2017). A CNN architecture was proposed, taking the RGB frame and a set of neighboring frames from the same video as input and identifying which of the neighboring frames, if any, contain the object being looked at and the coordinates of the human gaze.

Even though gaze estimation and human attention have been extensively studied, few works have integrated human attention with target object prediction. Among these few, Saran et al. (2018) proposed an approach to predict the human referential gaze, having both the person and object of attention visible in the image. The proposed network contains two pathways: one estimating the head direction and another for salient objects in the scene. Such a network was used as a backbone by Chong et al. (2020). In the latter, differently from Saran et al. (2018), an LSTM-based spatio-temporal model is used to leverage the temporal coherence of video frames to improve gaze direction estimation. However, only the direction of human gaze is predicted by Chong et al. (2020), while the information about the target object is not provided.

In this report, we adapt the LSTM-based spatio-temporal model from Chong et al. (2020) to an HRI setting, specifically a table-top scenario where the robot and human partner are positioned on opposite sides of a table, with the human and objects within the robot’s field of view. We fine-tune the model using the proposed *ObjectAttention* dataset, which is annotated with both object bounding boxes and the gazed target object. Additionally, we integrate it with human pose estimation and a face detector to enable real-time processing on the iCub robot. Using human pose estimation alongside an RGB-based face detector rather than an eye-tracking system was motivated by our commitment to have a natural HRI. Furthermore, studies suggested that humans shift their gaze, moving first the head and then the eyeballs in a linear and coordinated way, known as eye-head coordination (Maesako and Koike, 1993; Melvill Jones et al., 1988). Such eye-head temporal coordination especially characterizes conscious situations (contrarily, situations in which eyes precede the head movements are processed at an unconscious level) (Doshi and Trivedi, 2009). Finally, by integrating an online object detection method, we allow the system to predict the class label and location of the gaze target object. Note that, unlike Saran et al. (2018), by using Maiettini et al. (2019b) for object detection, the entire system can be easily adapted to detect novel target objects in only a few seconds. All the mentioned improvements result in an online robotic application that makes the robot capable of inferring where the human partner’s attention is targeted while interacting with them.

## 3 Methods

**FIGURE 1:**
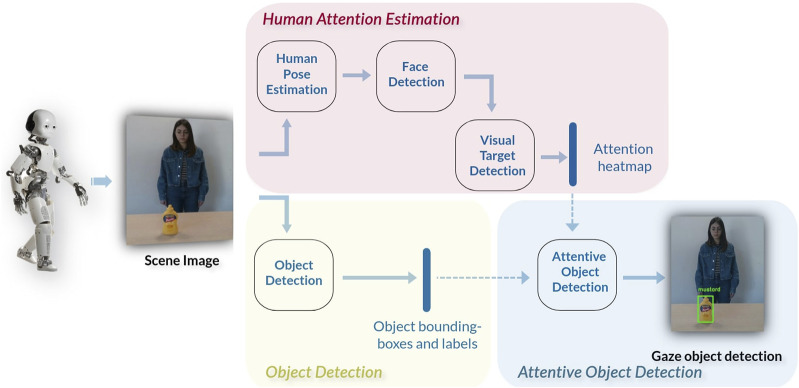
Pipeline of the presented architecture. The *Human Attention Estimation* pathway produces a heatmap of the gaze target. This heatmap and the bounding boxes and labels from the *Object Detection* module are then used by the *Attentive Object Detection* module to predict the specific object that is visually attended by the human.

The proposed pipeline is made of three pathways (Figure 1): the *Human Attention Estimation* pathway aiming at detecting the attention target of the human, the *Object Detection* pathway that recognizes and localizes the objects in the scene, and the *Attentive Object Detection* pathway that provides the gazed object from the human.

### 3.1 Human attention estimation

The *Human Attention Estimation* pathway has three distinct modules: 1) Human Pose Estimation, 2) Face Detection, and 3) Visual Target Detection. Having the RGB image as input, the final output of this pathway is the real-time prediction of the human attention target, provided as a heatmap.

#### 3.1.1 Human pose estimation

We rely on the OpenPose architecture proposed by Cao et al. (2019). In brief, OpenPose is a system for multi-human pose estimation that receives as input RGB frames and predicts the location in pixel (x,y) of 135 anatomical keypoints of each person in the image. It also associates a confidence level k to each prediction. The choice of *Human Pose Estimation* is motivated by having access to anatomical keypoints, facilitating further applications such as action recognition.

#### 3.1.2 Face detection

We rely on the face recognition presented by Lombardi et al. (2022b) to detect and extract the human face from the image. Specifically, the face keypoints extracted by the *Human Pose Estimation* module are used as input, while the output is the bounding box of the person’s head in front of the robot. Note that Chong et al. (2020) assume that the information of the face location is available. That is a strong limitation in applying the method in online robotic applications, preventing it from being used on real robots. In this work, we provide the online input to the *Visual Target Detection* module by using *Human Pose Estimation* together with *Face Detection*, enabling the pipeline to operate on the actual robot.

#### 3.1.3 Visual target detection

This module takes as input the RGB image from the robot camera and the human face bounding box extracted by the *Face Detection* module. It provides as output the heatmap representing the image area that more likely contains the target of human attention. Specifically, this is an image-sized matrix where each cell corresponds to an image pixel. The value of each cell ranges from 0 to 1 (respectively, the lowest and the highest probability to be –or to be close to– the target of human attention). For this module, we rely on the network presented by Chong et al. (2020), which is composed of three main parts. The first one is the *Head Conditioning Branch*, which uses the head bounding box encoded into a convolutional feature map (head feature map) together with the information of the location of the human’s head in the image to predict a first attention map. The second part is the *Main Scene Branch*, which multiplies the convolutional feature map of the entire image with the attention map and concatenates the result with the previously computed head feature map. The final tensor represents the input for the third and last part, namely, the *Recurrent Attention Prediction Branch*. This first encodes the tensor used as input for a convolutional long short-term memory network, then creates the final attention heatmap by upsampling the latter’s output using a decoder. In this work, we fine-tune the network’s weights using our dataset, and the resulting model is used for the developed application and the experimental analysis.

### 3.2 Object detection

The *Object Detection* pathway is characterized by one module that takes the RGB images from the robot’s camera as input and outputs the bounding boxes of all the objects of interest present in the scene. For this task, we rely on the online object detection approach presented by Ceola et al. (2021) and Maiettini et al. (2019a). This Mask R-CNN-based system is easily retrainable online, ensuring swift adaptation without compromising performance. We train the online object detection with data acquired using the pipeline described by Maiettini et al. (2017).

### 3.3 Attentive object detection

The third pathway combines the extracted information from human attention with the objects in the scene to detect the object that is the target of the human gaze. It takes as input the RGB image, the heatmap from the *Visual Target Detection* module, and all the bounding boxes and labels predicted by the *Object Detection* pathway. The output is the attended object bounding box and label.

Initially, the heatmap undergoes thresholding to isolate the region with values surpassing a refined threshold (the hottest part of the heatmap). This process aims to pinpoint the area indicative of human gaze focus within the image. Then, we compute the center of the obtained area and the surrounding bounding box. We use this information to select the object that is the most likely focus of human attention. Precisely, we choose the object that either presents a higher value of intersection over union (IoU) with the bounding box of the hottest part of the heatmap or, if this latter does not intersect any object bounding box, we select the object whose center is the closest to the center of the hottest part.

## 4 Dataset

A major contribution of this work is the *ObjectAttention* dataset. It depicts HRIs in a table-top scenario where the human gazes at different objects, and the robot understands the gaze direction and the target object.

### 4.1 Data collection

We recruited 10 participants (four women and six men) with normal or corrected vision (six people wore glasses). Data collection was conducted with the iCub robot (Metta et al., 2010), and all participants provided written informed consent. To collect the dataset, the iCub was positioned on one side of a table, with a RealSense 415 camera^1^mounted on its head. We placed up to five objects from the YCB dataset (Calli et al., 2015) on the table in various arrangements. The layout and object mix were different for each participant. The participants were instructed to stand on the other side of the table, facing the robot and looking at the requested object in a natural and spontaneous manner. The frames were recorded using the RealSense 415 camera and the YARP middleware (Metta et al., 2006).

We collected data in five sessions with each participant, starting with one object in the scene and gradually increasing the number of objects up to five. We performed two trials for each session, keeping the same number of objects but changing the object types and their arrangements on the table. For each session and trial, we collected a 5 s video for each different object, annotating the gazed target object as ground truth.

The resulting dataset consists of 250 videos (22,732 frames) depicting 10 participants in two different trials for each of the five sessions, gazing at the different objects. Additionally, for at least one trial per session, we placed a distracting object (i.e., the *Pringles* object) on the table, at which the participant was not asked to gaze. Details and example frames are reported in the Supplementary Material.

**FIGURE 2:**
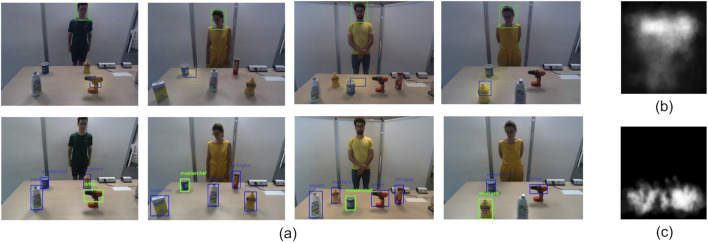
**(A)** Selection of sample output frames of the proposed pipeline. The first row depicts the scene image, as well as the head bounding box of the participant detected by the *Face Detection* module, the attention heatmap of the participant, and the bounding box of the hottest area of the heatmap. The second row depicts the related gaze target selections for the frames of the first row. **(B)** Gaze target location density for the dataset used in Chong et al. (2020) and **(C)** gaze target location density for the *ObjectAttention* dataset.

Finally, our motivation to collect a new dataset is that the dataset of Chong et al. (2020) contains more conditions in which the gaze was directed toward the upper part of the map (not suitable for a table-top). Our dataset, used to fine-tune the learning model, was collected for scenarios where the human and the robot look at objects placed on a table. Figure 2 depicts the density map of the gaze targets for the dataset in Chong et al. (2020) (b) and the one we collected (c).

### 4.2 Data annotation

Each setting requires bounding boxes for the participant’s head and the target object. The participants’ bounding box was extracted using the keypoints estimated by *Openpose* (Cao et al., 2017) and manually refined to be considered as ground truth. Furthermore, we manually annotated the bounding boxes and classes for all the objects on the table, highlighting the one that is the target of the human’s attention. The gaze target point was chosen as the center of the gazed object. The bounding box labeling was done using the *LabelImg* ^2^ framework.

## 5 Experiments

### 5.1 Model training

Both the *Object Detection* and the *Visual Target Detection* modules were re-trained to better suit the considered conditions.

#### 5.1.1 Object detection training

We trained the online object detection with data acquired using the pipeline described in Maiettini et al. (2017). Specifically, a human teacher showed the objects of interest to the robot, one at a time, holding them in their hand and moving them in front of the robot for approximately 30 s. The information from the robot’s depth sensors was used to localize the object and follow it with the robot’s gaze. The latter can be segmented, and the corresponding bounding box was automatically assigned through a depth segmentation routine (i.e., the learning object is the closest to the robot’s camera) and gathered as ground truth together with the object’s label, provided verbally. After each object demonstration, the collected data were used to update the current object detection model.

#### 5.1.2 Visual target detection fine-tuning

To fine-tune the *Visual Target Detection* module, we randomly split the *ObjectAttention* dataset by participants, considering approximately 70% of the dataset (data from seven participants) as a training set, and the remaining 30% as a test set, ensuring no overlap of data between the train and test splits. We fine-tuned the spatio-temporal model of the *Visual Target Detection* module on the training set, performing a warm training re-start with the pre-trained weights provided by the authors Chong et al. (2020) and empirically choosing the hyper-parameters as follows: *learning rate* $=5e^{-5}$ , *batch size* =4 , *chunk size* =3 , *number of epochs* =10 . To ensure the statistical relevance of the presented experiments, we repeated the training and evaluation of the model three times with three different splits of the dataset.

### 5.2 Experimental setup

The performance of the *Visual Target Detection* module is evaluated in terms of the *area under the curve* (*AUC*) and *Distance* metrics. For the AUC, each cell in the spatially discretized image is classified as either the gaze target or not. The ground truth comes from thresholding a Gaussian confidence mask centered at the human annotator’s target location. The final heatmap provides the prediction confidence score evaluated at different thresholds in the ROC curve. The *AUC* of this ROC curve is considered. The *Distance* metric is defined as the ${Ł}_{2}$ distance between the annotated target location and the prediction given by the pixel of the maximum value in the heatmap, with image width and height normalized to 1. The performance for the entire pipeline is measured in terms of the *Accuracy* of the detected gazed objects. For each image, the bounding box of the predicted gazed object is compared with the ground truth: if the gazed object is correctly identified, the prediction is counted as a true positive; otherwise, it is considered a false negative.

### 5.3 Visual target detection fine-tuning

First, we analyze the impact of fine-tuning the *Visual Target Detection* module on our dataset. In Table 1, we report the performance comparison of the proposed model (row **Fine-tuned model**) with the model presented in Chong et al. (2020) (row **Pre-trained model**) in terms of mean and standard deviation over the three dataset splits mentioned above. As can be seen, the fine-tuned model reports better performance on the proposed *ObjectAttention* dataset. Specifically, the predicted hottest point in the heatmap is closer to the true gazed point of ∼ 0.04. Note that this is a relevant difference because the *Distance* metric is computed on an image with width and height normalized to 1. This result is also supported by the improvement in the *AUC* of 5% . To quantify the distance metric in the task space, we used the depth information and the intrinsic camera parameters to calculate the Euclidean distance between the 3D coordinates of the center of the ground truth bounding box of the gaze target object and the center of the predicted bounding box of the gaze target object. It results in a task space distance of 0.092±0.127 meters.

**TABLE 1:** Quantitative evaluation of the *Visual Target Detection* model on the presented *ObjectAttention* dataset.

Method	AUC (%) ↑	${Ł}_{2}$ distance ↓
Pre-trained model	87.5 ± 0.9	0.131 ± 0.014
Fine-tuned model	92.5 ± 1.9	0.089 ± 0.014

The enhanced performance stems from fine-tuning the model with a dataset more aligned with the target scenario (table-top). Nevertheless, because the fine-tuned network has been initialized with the weights presented in Chong et al. (2020), the final model can predict gaze directions that differ from those considered in the proposed dataset (see the video provided as Supplementary Material).

### 5.4 Accuracy evaluation

In order to evaluate the performance of the overall pipeline, we choose one of the models trained on the three different train/test splits and use it in our pipeline. Quantitative results are obtained using the same test set previously employed for evaluating the fine-tuned model, with ground truth provided by bounding boxes and labels of objects on the table and the target gazed object.

First, we analyze the overall accuracy of the pipeline in detecting the gazed target object of the three different participants in the test set. Our experiments indicate a success rate of 79.5% in correctly detecting the target object. This number reflects the integrated performance of the *Visual Target Detection*, *Object Detection*, and *Attentive Object Detection* modules.

Figure 2 illustrates a selection of sample frames from the output, including attention heatmaps and bounding boxes, highlighting the head of the participant (detected by the *Face Detection* module) and the hottest areas of the heatmap in the frames of the top row while the final gaze object bounding box and label are presented in the bottom row frames (see also in the video in the Supplementary Material).

With the aim to be in line with the current state-of-the-art, we benchmarked a visual language model (VLM) to evaluate the overall accuracy. We choose the open-source LLAVA-1.6 model as the VLM (Liu et al., 2024), which reports a success rate of 15% in correctly detecting the gazed object. The very poor performance is explained by the fact that a VLM is not targeted to solve a highly specific task like the one reported in this report. More details are reported in the Supplementary Material.

### 5.5 Performance analysis

#### 5.5.1 Per object performance

**FIGURE 3:**
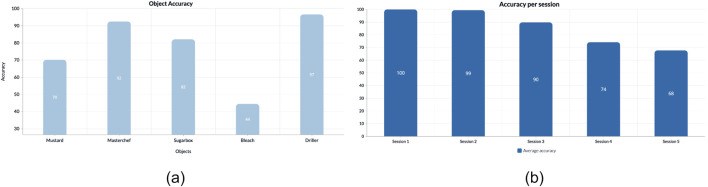
Accuracy analysis: **(A)** on each of the objects involved in the experiments and **(B)** on each session for all participants. The session number also reflects the corresponding number of objects present in the scene.

In Figure 3B, we present the achieved accuracy levels for various target objects. The system demonstrates high performance across most objects, except for the *Bleach* class. This discrepancy arises from challenges in object detection, leading to occasional inaccuracies in locating the *Bleach* object. Such issues may result from disparities between the detector’s training conditions and the testing environment, indicating a domain shift. Previous studies have suggested addressing this issue through methods such as integrating autonomous exploration by robots in new domains and employing weakly supervised learning techniques (Maiettini et al., 2019b; Maiettini et al., 2021).

#### 5.5.2 Per session performance

Figure 3A depicts the accuracy levels of the overall pipeline in various sessions. The performance of the system slightly decreases for higher numbers of sessions. This is reasonable because, in those cases, the number of objects increases; thus, the table becomes more cluttered. However, the accuracy level is still acceptable (around 70%) even with the most cluttered scenes, showing that this is not a limitation of the proposed system.

#### 5.5.3 Distractors

We investigate the impact of distracting objects on system performance, selecting a sample object (i.e., *Pringles*) as a distractor. Although participants were not instructed to focus on this object, our *Object Detection* module is trained to detect it. Our objective is to assess whether the presence of the distractor hinders the accurate identification of the target object. The results indicate that prediction errors occur in only about ∼ 3% of frames with the distracting object, suggesting it is not a significant limitation.

#### 5.5.4 Distance-based performance

A further analysis was conducted to evaluate the system’s accuracy while systematically varying the distance between objects from 0 cm to 100 cm. Our method achieved 74% accuracy at 0 cm and over 98% accuracy when objects were separated by more than 60 cm. The Supplementary Material provides more details.

#### 5.5.5 Real-time feed performance

The Chong et al. (2020) architecture, initially burdened by high latency due to reloading the model for each input frame, resulted in less than 5 fps output speed when integrated with our proposed system. To improve real-time performance, we separated model initialization from the code, initializing it only once. This adjustment boosted the output frame rate to 8 fps, deemed experimentally sufficient as the humanoid iCub’s dynamics are slower than the camera frame rate.

#### 5.5.6 Edge case performance

We assess the robustness of our system by conducting experiments on edge cases, including scenarios where the human partner is positioned at an angle relative to the robot and objects placed on the line of sight. These experiments yielded an overall accuracy level of 75% . For more details, refer to the Supplementary Material.

## 6 Conclusion

We presented a learning system for detecting human attention toward objects in the scene. Our method combined an online object detection algorithm with a network for gaze estimation conditioned on the estimation of the human pose. We demonstrated its effectiveness through an extensive experimental analysis using the iCub robot. Our results indicated that integrating face detection, human attention prediction, and online object detection in our pipeline enables the robot to perceive and interpret human gaze within its environment. Such an achievement promises to enhance the robot’s social awareness and responsiveness, allowing for more natural interactions in social robotics, which makes it well-suited to be used in applications such as assistant tutoring, robot-assisted therapies, and interaction with children with autism spectrum disorder (Alabdulkareem et al., 2022; Yousif, 2020; Calderita et al., 2014). The pipeline and dataset presented establish the foundation for our ongoing efforts to enhance iCub’s collaborative task capabilities by integrating diverse social cues within a multimodal architecture. Our forthcoming endeavors will focus on integrating a segmentation layer to optimize system performance in more complex scenes (e.g., highly cluttered scenarios or the presence of non-convex objects). Another considered direction is to include out-of-frame target detection to identify when humans are not focused on the preferred task.

## Data Availability

The raw data supporting the conclusions of this article will be made available by the authors, without undue reservation. The code, learning models, and dataset can be found at https://github.com/hsp-iit/online-attentive-object-detection.

## Footnotes

1
https://www.intelrealsense.com/depth-camera-d415/

2
https://github.com/tzutalin/labelImg
