# Supplementary material for: A pipeline for estimating human attention toward objects with on-board cameras on the iCub humanoid robot
Source: Front Robot AI. 2024 Oct 17;11:1346714. doi: 10.3389/frobt.2024.1346714 (PMC11524796; doi:10.3389/frobt.2024.1346714)
Supplement: Supplementary file 1 [file DataSheet1.pdf]

Supplementary Material

1 EXPERIMENTAL SETUP

The ObjectAttention dataset collection and experimental work involve the participation of the humanoid robot, iCub, in a tabletop scenario, as depicted in Fig.S1. While 6 distinct objects from the YCB dataset Calli et al. (2015) are placed on the table in different settings, the human partner is asked to stand on the opposite side of the table gazing at each of the objects.

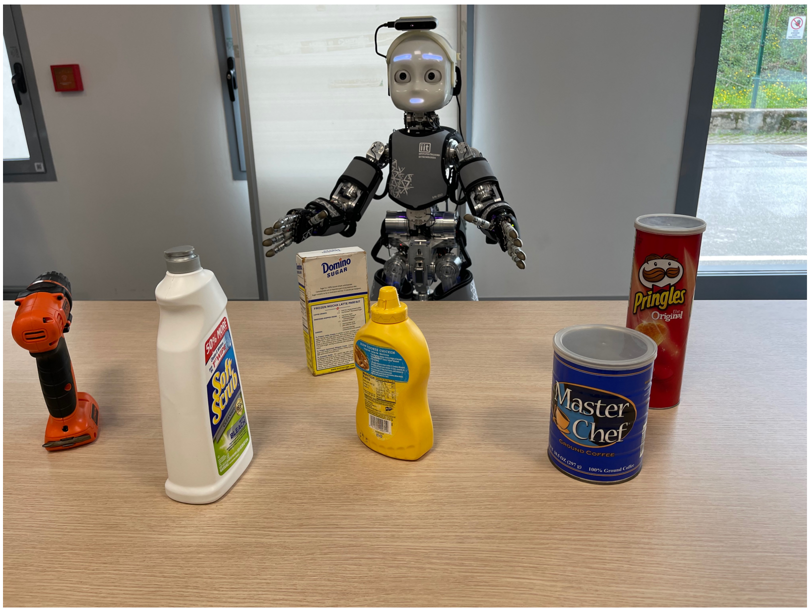

Figure S1: *ObjectAttention* dataset: data collection setup with 6 objects from the YCB dataset Calli et al. (2015) placed on a table between a human partner and iCub. Objects from left to right: *Driller, Bleach, Sugarbox, Mustard, MasterChef, Pringles*

2 DATASET

The ObjectAttention dataset is one of the contributions of this study. It is collected from 10 participants, in 5 different sessions and settings. The objects are placed in random configurations and the participants are asked to gaze at each of the objects for a period of 5 seconds. While each frame can be considered separately as depicted in Fig.S2, it is also viable to view sequences of frames capturing the gaze focused on each object as video clips.

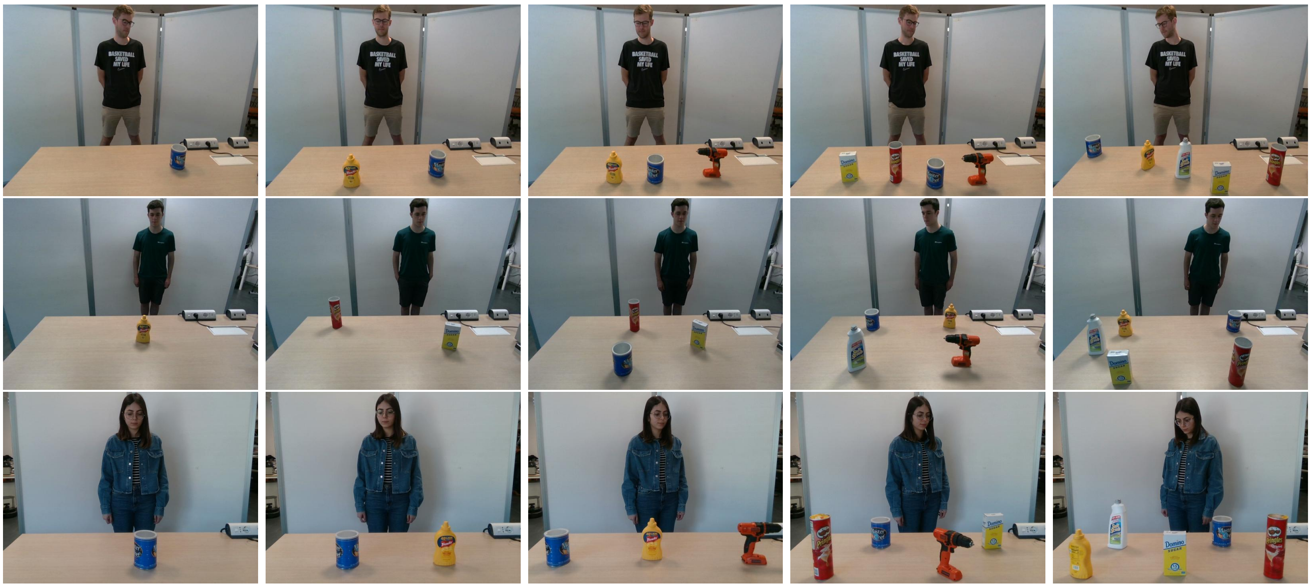

Figure S2: Sample frames extracted from the proposed *ObjectAttention* dataset, representing different participants performing the gazing task, over five sessions with different number of objects involved.

3 DISTANCE-BASED PERFORMANCE

To assess the effectiveness of our proposed architecture in discerning object distances on a table, we conduct supplementary experiments involving three participants (comprising two females and one male) and utilizing two objects sourced from the YCB dataset Calli et al. (2015). Beginning with the objects positioned at a distance of $0cm$, we systematically increase the gap by $20cm$ intervals up to $100cm$. Fig.S4 depicts sample frames of the setup for this experiment. At each interval, the system's output for each object is recorded. Notably, for comprehensive analysis, we replicate experiments at each distance point across three distinct directions. The accuracy reported in Fig. S3 represents the average accuracy across different directions at specific distances. With an accuracy level of 70% observed at a 0 centimeter distance between objects, escalating to over 98% when the distance between objects exceeds 60 centimeters, our findings suggest that our proposed architecture exhibits robust performance regardless of the proximity of objects.

4 EDGE CASES PERFORMANCE

To further analyze the performance of our proposed architecture, we collected a supplementary set of data including edge cases, such as positioning the human partner at an angle to the robot and placing the objects in the line of sight. Data collection involved 3 participants (2 females, 1 male), over 5 distinct sessions. Six objects from the YCB Calli et al. (2015) dataset were used, with the number of objects in the scenes gradually increasing throughout the sessions. Performance was evaluated in each session under two different settings, each featuring varying objects, placements, and human partner positioning.

The experiments conducted on this set of data resulted in the overall accuracy of 75%. More specifically, level of 78% for angled human positioning, and 73% for objects in the line of sight, accuracy values that are in line with the performance reported in the main text on the testset split derived from the ObjectAttention dataset. Fig. S5 depicts samples from the edge cases including human partner positioned at an angle and objects placed on the line of sight.

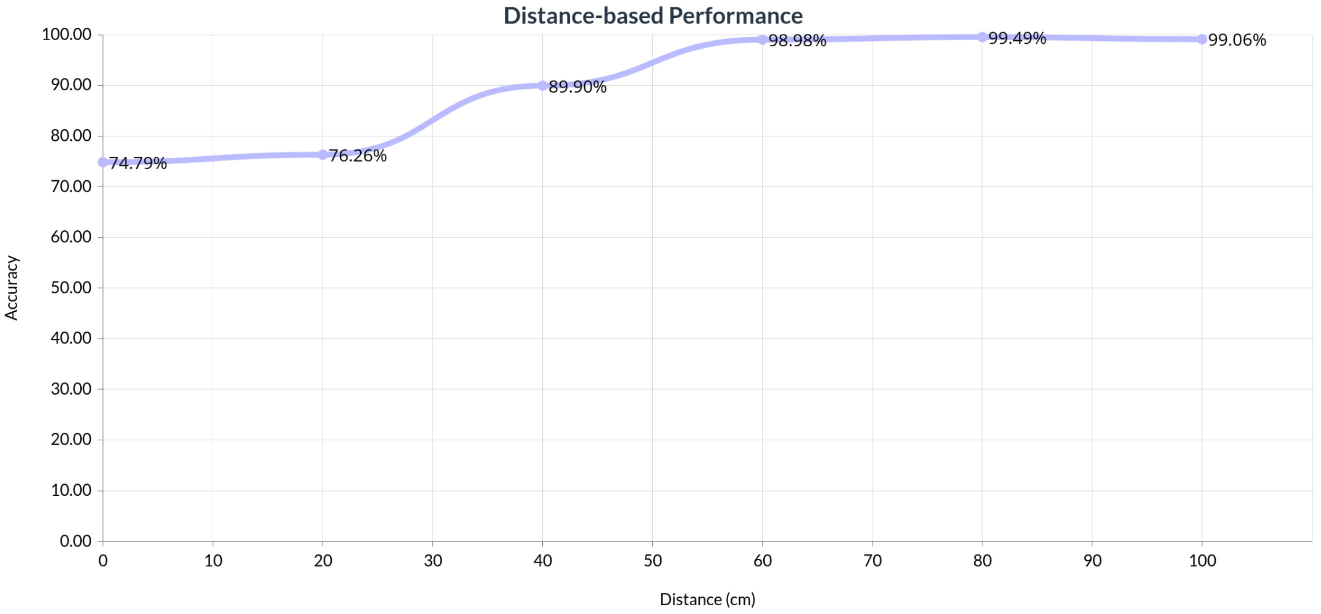

Figure S3: Distance-based performance evaluation. The proposed architecture demonstrates 74.79% accuracy when objects are at a $0cm$ distance, increasing to over 98% accuracy when the distance between objects exceeds $60cm$.

5 VISUAL LANGUAGE MODEL BENCHMARK

With the aim to be inline with the current state-of-the-art, we benchmarked a Visual Languange Model (VLM) on our ObjectAttention dataset in order to evaluate the overall accuracy in predicting the object gazed by the human. Precisely we choose the open source LLAVA-1.6 model as VLM Liu et al. (2024). In order to make LLAVA able to detect the objects of interest in a precise manner, we wrote the textual prompt in input to the model using different labels respect to those used in the YCB dataset. The chosen prompt was the following: *Which object is the person looking at? 1. white bottle, 2. yellow bottle, 3. blue round can, 4. red tube, 5. box, 6. driller? Choose ONLY one option answering ONLY with 1,2,3,4,5,6*. Then, the output was converted in one of the YCB labels: bleach (white bottle), yellow bottle (mustard), blue round can (masterchef), red tube (pringles), box (sugarbox), driller (driller).

LLAVA model was run on the same testset used to evaluate the proposed fine-tuned model, reporting a success rate of 15% in correctly detecting the gazed object. The very poor performance is explained by the fact that a VLM with its general capabilities is not targeted to solve highly-specific task like the one reported in this report (probably because the VLM training data do not include enough classification data and cover enough classes).

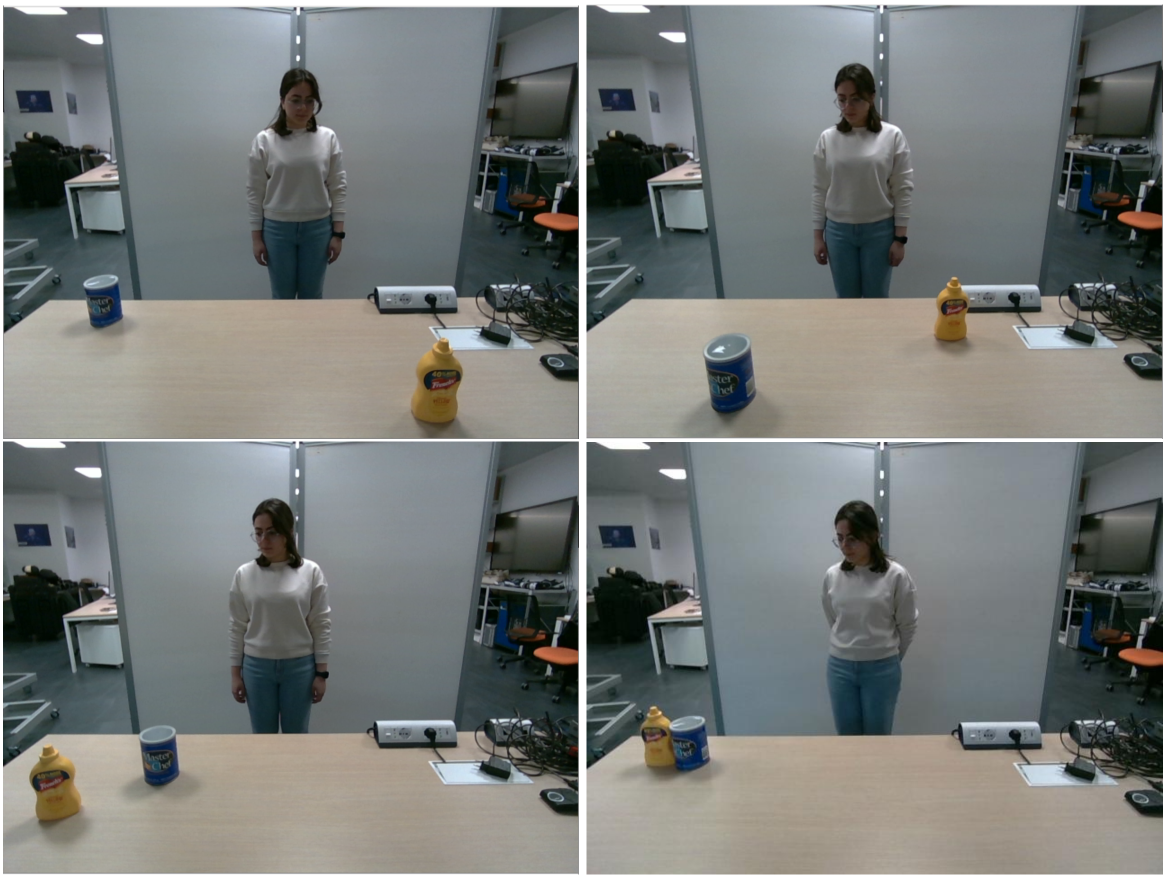

Figure S4: Sample frames extracted from collected data for distance-based performance evaluation, illustrating objects positioned at various distances.

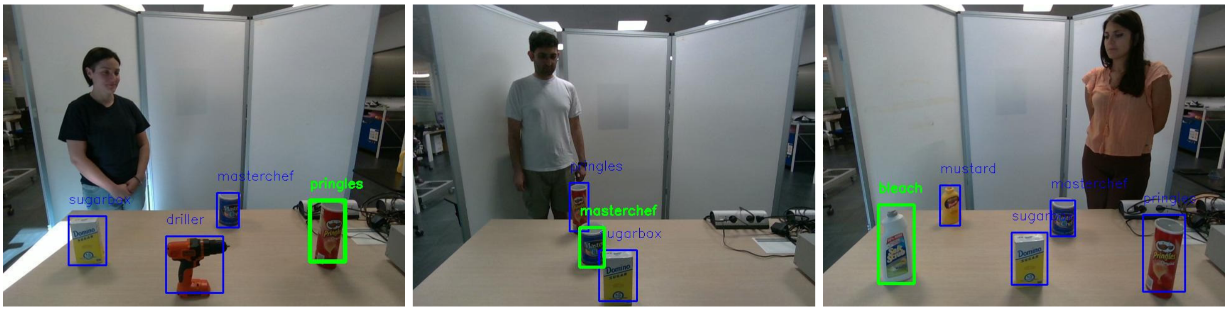

Figure S5: Sample frames from supplementary data for edge cases. Includes samples with human partner positioned at an angle to robot and objects placed on the line of sight.

REFERENCES

Calli, B., Singh, A., Walsman, A., Srinivasa, S., Abbeel, P., and Dollar, A. M. (2015). The ycb object and model set: Towards common benchmarks for manipulation research. In *2015 international conference on advanced robotics (ICAR)* (IEEE), 510–517

Liu, H., Li, C., Wu, Q., and Lee, Y. J. (2024). Visual instruction tuning. *Advances in neural information processing systems* 36
